# Metformin Results in Diametrically Opposed Effects by Targeting Non-Stem Cancer Cells but Protecting Cancer Stem Cells in Head and Neck Squamous Cell Carcinoma

**DOI:** 10.3390/ijms20010193

**Published:** 2019-01-07

**Authors:** Selena Z. Kuo, Christine O. Honda, Wei Tse Li, Thomas K. Honda, Elizabeth Kim, Xabier Altuna, Eric Abhold, Jessica Wang-Rodriguez, Weg M. Ongkeko

**Affiliations:** 1 Department of Otolaryngology-Head and Neck Surgery, University of California, San Diego, CA 92093, USA; selenazkuo@gmail.com (S.Z.K.); christinehonda70@gmail.com (C.O.H.); wtl008@ucsd.edu (W.T.L.); thomashonda@sandiego.edu (T.K.H.); elizabethkim2011@gmail.com (E.K.); eabhold@gmail.com (E.A.); 2 Hospital Donosita, 20014 Donostia, Gipuzkoa, Spain; XABIER.ALTUNAMARIEZCURRENA@osakidetza.net; 3 Veterans Administration Medical Center and Department of Pathology, University of California, San Diego, CA 92092, USA; jessica.wang-rodriguez@va.gov

**Keywords:** metformin, cancer stem cells, head and neck squamous cell carcinoma, reactive oxygen species

## Abstract

Cancer stem cells (CSCs) have been shown as a distinct population of cancer cells strongly implicated with resistance to conventional chemotherapy. Metformin, the most widely prescribed drug for diabetes, was reported to target cancer stem cells in various cancers. In this study, we sought to determine the effects of metformin on head and neck squamous cell carcinoma (HNSCC). CSCs and non-stem HNSCC cells were treated with metformin and cisplatin alone, and in combination, and cell proliferation levels were measured through MTS assays. Next, potential targets of metformin were explored through computational small molecule binding analysis. In contrast to the reported effects of metformin on CSCs in other cancers, our data suggests that metformin protects HNSCC CSCs against cisplatin in vitro. Treatment with metformin resulted in a dose-dependent induction of the stem cell genes CD44, BMI-1, OCT-4, and NANOG. On the other hand, we observed that metformin successfully decreased the proliferation of non-stem HNSCC cells. Computational drug–protein interaction analysis revealed mitochondrial complex III to be a likely target of metformin. Based on our results, we present the novel hypothesis that metformin targets complex III to reduce reactive oxygen species (ROS) levels, leading to the differential effects observed on non-stem cancer cells and CSCs.

## 1. Introduction

Metformin is a biguande class compound that is the most widely prescribed and well-tolerated drug for type II diabetes. In recent years, it has also received attention as a potential anticancer agent. A retrospective study done by Evan et. al. was the first to show a correlation between metformin treatment for diabetes and a decreased risk of various cancer types [1]. Furthermore, a more recent clinical study reported that metformin improves response to chemotherapy in breast cancer patients with diabetes [2]. The ability of metformin to act as an anticancer drug has been attributed to both the indirect effects of lowered insulin levels and the direct targeting of tumor cells. Hirsch et al. demonstrate that metformin, at clinically relevant doses, is able to preferentially target breast cancer stem cells (CSCs) [3]. CSCs are a small subpopulation of cells within a tumor that share the stem cell capabilities of self-renewal and differentiation. Unlike the majority of cancer cells, these stem cells are resistant to conventional chemotherapy treatment and can regenerate tumors, which can lead to relapse of disease. Therefore, drugs that directly target CSCs offer substantial promise for the complete eradication of a tumor.

In order to effectively target both non-stem and stem cell populations, drugs that selectively target cancer stem cells are often tested in combination with conventional chemotherapy treatments that can deplete the bulk of the tumor. The in vivo studies of Hirsch et. al. revealed that low doses of metformin in combination with doxorubicin synergistically reduced tumor mass and prolonged remission in xenograft mouse models more effectively than either drug alone [3]. In other studies, metformin showed comparable synergy with paclitaxel and carboplatin in breast, lung, and prostate cancer [4]. In ovarian cancer stem cells, metformin was able to decrease cellular proliferation and increase the cytotoxic effects of cisplatin both in vitro and in vivo [5].

Metformin’s mechanism of action has mainly been attributed to the activation of AMP-activated protein kinase (AMPK) and the inhibition of Complex I of the mitochondria [6]. AMPK inhibits the mammalian target of rapamycin (mTOR), a key regulator of cell growth and proliferation [7]. In HNSCC, mutations in the Akt/mTOR pathway are considered some of the strongest oncogenic drivers [8], which makes metformin an attractive therapeutic agent to study. Several other targets of metformin, such as mitochondrial glycerophosphate dehydrogenase (mGPD) and ataxia telangiectasia mutated (ATM), have been proposed and validated to some degree, suggesting that metformin’s mechanism is complex and multi-faceted [6]. Despite multiple hypotheses of metformin action, many aspects of its physiological effects, such as the alteration of gut microbiome composition, are not well understood [6]. Furthermore, there are few molecular studies that validate the binding of metformin to proposed targets. For example, although metformin has been observed to inhibit complex I of the mitochondria in multiple studies, no definitive mechanism of the inhibition exists, and multiple groups claimed that they could not obtain direct evidence of complex I inhibition [9].

In this study, we sought to determine the effects of metformin on HNSCC CSCs and non-stem cell cancer cells. In contrast to the reported effects of metformin on CSCs of other cancers, we demonstrated that metformin does not target HNSCC CSCs but instead promotes expression of stem cell markers, an indication of elevated stemness. Furthermore, when treated in combination with cisplatin, metformin significantly protected against chemotherapy-induced cell death. However, non-stem HNSCC cell populations were successfully reduced with metformin. We next investigated potential targets of metformin that could explain our results. Using a computational small molecule-to-protein docking software, we uncovered that the mitochondrial complex III interacts strongly with metformin. Since complex III is known as a major site of reactive oxygen species (ROS) production [10], and metformin has been demonstrated to reduce ROS levels in several studies [11,12], we offer the hypothesis that metformin reduces ROS levels through complex III inhibition to cause differential effects on HNSCC CSCs and non-stem cancer cells. Our hypothesis is supported by the fact that low ROS levels are required for CSC maintenance and self-renewal and that high ROS levels cause CSC differentiation or eradication [13,14]. On the other hand, an increased ROS level is a hallmark of non-stem cancer cells and is likely to be a driver of increased cancer cell proliferation and cancer progression [15,16]. The decrease in ROS levels mediated by metformin would promote stemness but reduce the ability of non-stem cancer cells to proliferate. Collectively, our results demonstrate that metformin is not effective for treating HNSCC in combination with cisplatin but could be an attractive treatment option if a third drug is used to selectively reverse the ROS-reducing effects of metformin in CSCs.

## 2. Results

### 2.1. Metformin Mitigates Cisplatin-Mediated Cell Death in HNSCC CSCs but Reduces Cell Proliferation in Non-Stem HNSCC Cells

**Figure 1:**
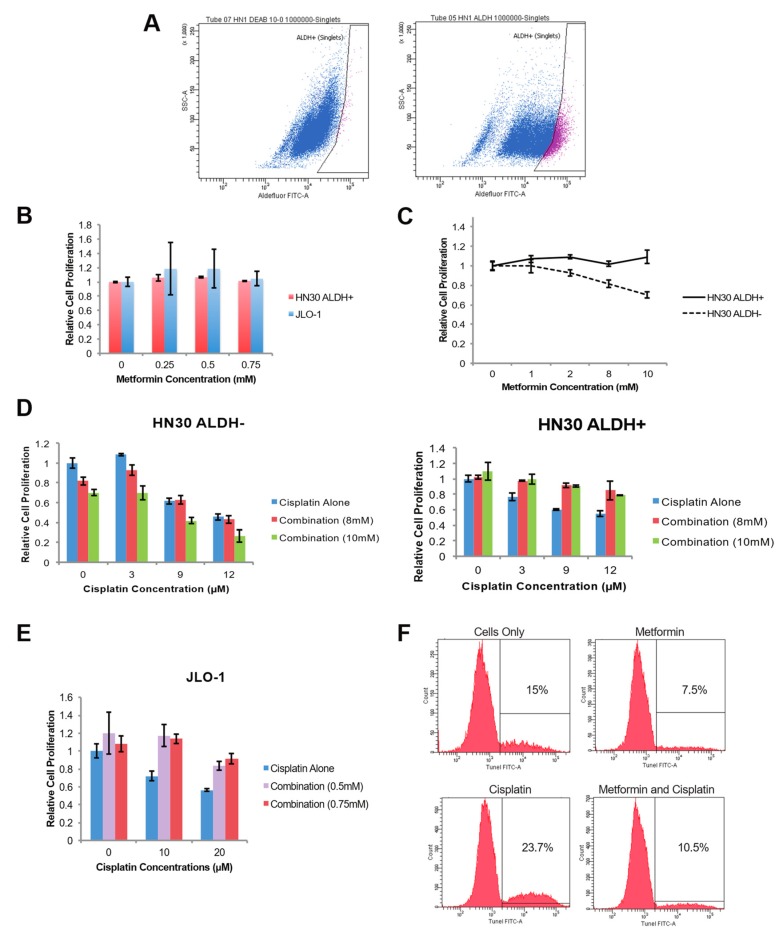
Metformin’s effects on head and neck squamous cell carcinoma (HNSCC) cancer stem cells (CSCs) and non-stem HNSCC cells. (**A**). Flow cytometry sorting of ALDH− and ALDH+ cells from the HN-30 cell line. (**B**). Cell proliferation levels of ALDH+ HN-30 cells and JLO-1 cells were measured through the MTS assay at 4 different concentrations of metformin applied. (**C**). Cell proliferation levels of ALDH+ HN-30 cells were compared against that of ALDH− HN-30 cells after application of metformin in various concentrations. (**D**). Cell proliferation levels of ALDH+ and ALDH− HN-30 cells after co-treatment of metformin and cisplatin. (**E**). Cell proliferation levels of JLO-1 cells after co-treatment of metformin and cisplatin. (**F**). TUNEL assay plots showing percentage of JLO-1 cells with DNA double strand breaks.

We have previously created the JLO-1 CSC cell line by isolating HNSCC cells from a fresh laryngeal tumor and culturing them under conditions that favored the growth of stem cells. Flow cytometry confirmed the culture to be almost entirely CD44+ compared to the nonspecific IgG antibody used as a control [17]. CD44 is an identified cell-surface marker for HNSCC stem cells, and in vivo studies showed that the enriched CD44+ population could give rise to new tumors while the CD44− population could not [18]. We have previously verified the stemness of JLO-1 by demonstrating significantly higher levels of aldehyde dehydrogenase (ALDH1), Oct-4, and Nanog compared to established HNSCC cell lines [17], and by demonstrating self-renewal. In addition, using the Aldefluor stem cell detection kit, we were able to sort the HNSCC cell line HN30 into ALDH+ and ALDH− populations using fluorescence-activated cell sorting (FACS) (Figure 1A). The ALDH marker isolates a subpopulation that is purer than using only CD44+ and has been utilized as a single-marker identifier of HNSCC CSCs [19].

Using the doses previously reported to target breast CSCs [20], MTS proliferation assays indicated that metformin alone has negligible effects on proliferation for both our putative CSC culture JLO-1 and the ALDH+ subpopulation of HN30 (Figure 1B). We next assessed the effectiveness of metformin on the separate ALDH+/− subpopulations. Using our FACS sorted populations, 72 h of metformin treatment decreased the proliferation of the ALDH- population, while causing little to no change in the ALDH+ fraction (Figure 1C). It is of note that only higher concentrations of metformin were able to produce a decrease in cellular proliferation in the non-stem cell ALDH− population.

Cisplatin is one of the most potent and commonly used chemotherapy drugs for treating head and neck cancer. However, our results indicate that metformin protects against the cytotoxic effects of cisplatin in both JLO-1 and the ALDH+ subpopulation of HN-30 (Figure 1D,E). At doses of 10 µM and 20 µM cisplatin, cell proliferation of JLO-1 decreased to 66% and 48% respectively (Figure 1E). However, even in combination with low doses of metformin, the cytotoxic effects of cisplatin were abrogated, and cell viability levels returned to values similar to the control (Figure 1E). A similar protection against cisplatin was not observed in the ALDH- population of HN30, and there was slight synergism between the two drugs at their respective highest concentrations (Figure 1D). Cisplatin causes cell death primarily through DNA crosslinking, so the effects of metformin on JLO-1 were further validated by measuring amounts of DNA strand breaks with a TUNEL assay. 20 µM of cisplatin causes 23.7% of the cells to undergo DNA strand breaks, while a combination treatment with 0.75 mM of metformin reduced the amount of DNA strand breaks to 10.5% (Figure 1F). To gain insight into a possible molecular mechanism behind the protective effect of metformin, we explored the well-studied cell survival pathway of Akt. Interestingly, our immunoblot showed a dose-dependent decrease of phosphorylated Akt in response to metformin treatment in JLO-1 cells, suggesting that metformin does not confer chemoprotective effects in CSCs through the Akt pathway (Figure S1).

### 2.2. Metformin Increases Stem Cell Characteristics in HNSCC CSC Population

**Figure 2:**
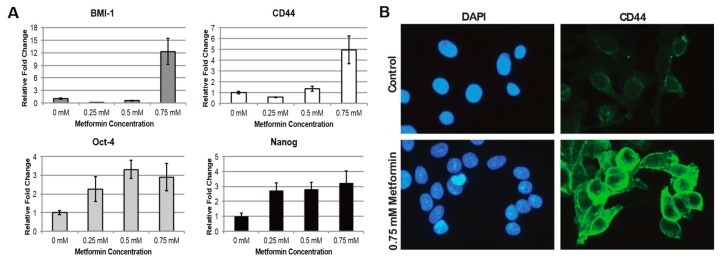
Metformin increases stem cell marker levels. (**A**). Plots of qPCR-measured gene expression level fold changes for stem cell markers after treatment of JLO-1 cells to different concentrations of metformin. (**B**). Immunofluorescence visualization of CD44 distribution on the cell surface before and after treatment with 0.75 mM metformin. The cell nuclei were stained with DAPI. All images were taken at 40× magnification.

To gain a more thorough understanding of metformin’s effects on HNSCC CSCs, we performed RT-qPCRs for JLO-1 cells to measure changes in expression of stem cell markers CD44, BMI-1, Oct-4, and Nanog after exposure to metformin. BMI-1 is a gene necessary for the stem cell property of self-renewal, and was shown to be differentially expressed in the CD44+ population in HNSCC [18]. Oct-4 and Nanog are transcription factors that are required to maintain pluripotency in embryonic stem cells [21]. Our results demonstrated an increase in all of the described stem cell genes, but most significantly in CD44 and BMI-1, where gene expression is increased up to 5 and 12-fold, respectively (Figure 2A). Interestingly, the strong expression increase is only observed at 0.75 mM of metformin applied, suggesting the existence of a possible threshold concentration for metformin to take effect. A verification of the increase in expression of CD44 was performed with immunofluorescence, in which the protein was observed to be highly localized to the cell surface (Figure 2B).

### 2.3. Computational Binding Analysis Reveals Strong Interaction of Metformin with Mitochondrial Complex III

**Figure 3:**
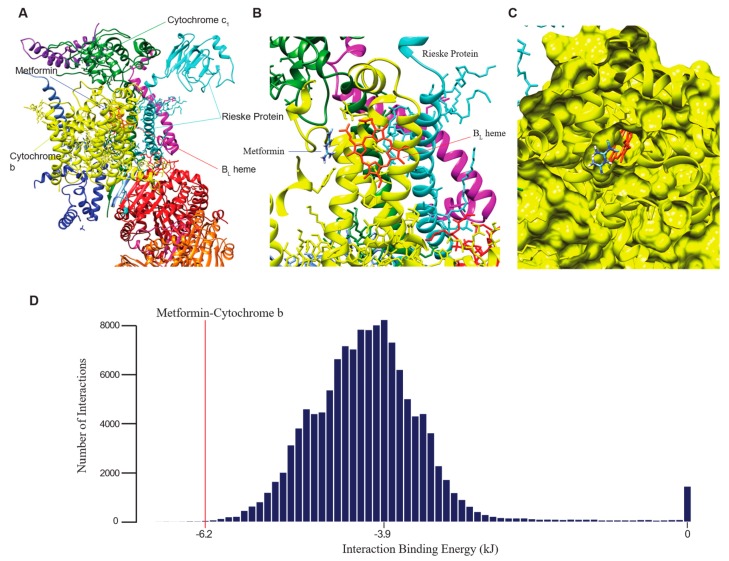
Computational prediction of complex III binding to metformin. (**A**). Broad-angle screenshot of UCSF Chimera’s visualization of metformin’s position of binding within complex III that is predicted to have the lowest energy. (**B**). Magnified view of metformin binding position. (**C**). Molecular surface visualization positions metformin within the same pocket as the B_L_ heme when it binds to complex III. (**D**). Plot of number of interactions vs. interaction energy of AutoDock Vina’s virtual screening of metformin’s interaction with 127,635 potential binding pockets on 250 proteins and their associated complexes. The red line indicates the predicted binding energy of metformin to the Q_o_ site of cytochrome b at −6.2 kJ.

To elucidate the mechanism of metformin action that could explain its observed effects in HNSCC, we used the small molecule-to-protein docking program AutoDock Vina to explore metformin’s binding interactions. Vina was demonstrated to be highly accurate in predicting position of binding and binding energies for small molecules that have low numbers of active rotatable bonds, such as metformin, which has two rotatable bonds [22]. We observed that metformin binds next to the B_L_ heme, near the Q_o_ site, of complex III with a binding energy of −6.2 kJ, suggesting high stability of binding (Figure 3A–C). The binding site is predicted to be within cytochrome b and on the side of the B_L_ heme away from the Rieske Protein (Figure 3B). From a molecular surface visualization of the binding, it can be seen that metformin and the B_L_ heme are located within the same pocket in cytochrome b (Figure 3C).

To validate that metformin binds more strongly to complex III at the proposed site than to other binding sites and that AutoDock Vina is capable of predicting a diverse range of binding interactions, we explored the binding possibility of metformin with the top 250 proteins, and any proteins that they complex with, that are most associated with high stemness in HNSCC tissue samples. The fpocket program was used to identify possible sites for small molecule to bind to these proteins, and a total of 127,635 putative binding sites were screened using AutoDock Vina for binding with metformin. The binding of metformin to complex III near the B_L_ heme is within the top 20 most stable interactions we found (Figure 3D). The mode binding energy to metformin of the binding sites screened is −3.9 kJ, whereas metformin binds to complex III with a binding energy of −6.2 kJ (Figure 3D). The lower the binding energy is, the more stable the interaction. Our results thus demonstrated that metformin binds to complex III with enough exclusivity to justify further investigation of complex III as a major target of metformin.

### 2.4. Expressions of Complex III Genes Correlate with Clinical Variables and Stem Cell Marker Expressions

**Figure 4:**
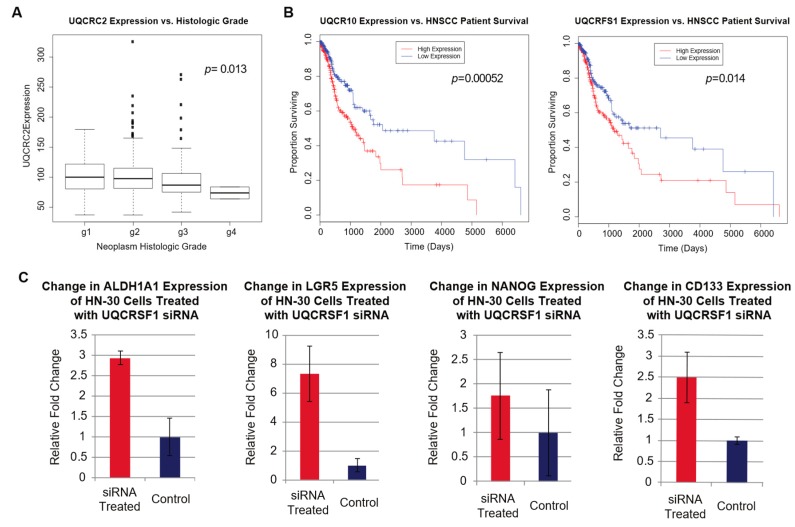
Correlation of complex III gene expressions with clinical variables and stem cell marker expressions. (**A**). Correlation of decreasing UQCRC2, one of the two core protein subunits of complex III, expression with increasing histologic grade of HNSCC patient samples using the Kruskal-Wallis test. (**B**). Correlation of UQCR10, a low-molecular weight protein subunit, and Rieske iron-sulfur protein (UQCRFS1) expressions with HNSCC patient survival. (**C**). Relative fold change of stem cell markers’ expressions after Rieske protein knockdown in HN-30 cells. UQCRC2 and UQCR10 are supporting subunits in the complex with no known reactive capabilities, while the Rieske protein is involved in the oxidation of ubiquinol.

Using gene expression data of HNSCC patient samples downloaded from The Cancer Genome Atlas (TCGA), we next correlated the expression of genes coding for complex III protein subunits to tumor histologic grade and patient survival to explore the clinical relevance of complex III activity. Interestingly, we discovered that high expressions of complex III genes correlated strongly with lower histologic grade but also lower patient survival, even though higher histologic grade suggests a more aggressive tumor and usually correlates with lower rate of survival (Figure 4A,B). Histologic grade is a measure of how well the tumors are differentiated, where a high grade indicates poor differentiation, and high histologic grade correlates strongly with the presence of CSCs [23,24]. Therefore, we interpret our result to suggest that lower complex III activity is associated with higher CSC presence. Since we have demonstrated that metformin increases stemness, we hypothesize that metformin’s interaction with complex III is inhibitory rather than activating. Following this hypothesis, we can further hypothesize that metformin’s inhibition of complex III decreases the proliferation of non-stem cancer cells. Our data are supportive of these hypotheses because the duration for which a patient can survive is correlated more strongly with cancer proliferation than with CSC presence, which is more associated with treatment resistance and tumor recurrence. Therefore, suppression of complex III would lead to a better survival rate by decreasing cancer cell proliferation but also lead to higher histologic grade by increasing the stemness of CSCs, which explains the strong correlation of low expressions of complex III genes to better survival prognosis and higher histologic grade at the same time.

**Figure 5:**
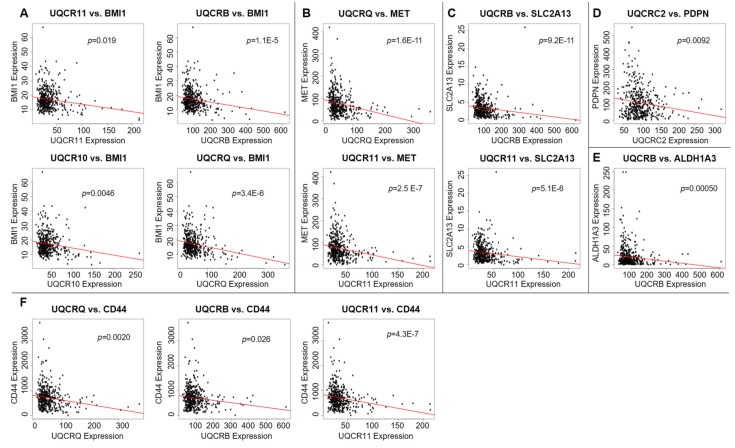
Scatter plots of complex III subunits’ expressions vs. expressions of various stem cell markers: (**A**) BMI-1, (**B**) MET, (**C**) SLC2A13, (**D**) PDPN, (**E**) ALDH1A3, and (**F**) CD44. Correlations were performed with Spearman’s correlation test (*p* < 0.05).

To validate our hypothesis that lower complex III activity is associated with higher CSC presence, we proceeded to explore the relationship between complex III and HNSCC stem cell markers. Using HN-30 cells, we knocked down expression of the Rieske protein subunit in complex III with siRNA and measured changes in the expression of HNSCC stem cell markers with RT-qPCR. We investigated eight of the most significant HNSCC stem cell markers described by Major et. al. [25], including NANOG, ALDH1A1, BMI-1, CD44, LGR5, CD133 (PROM1), ABCG2, and OCT-4 (POU5F1) (for primer sequences, see Table 1). We observed that the expressions of stem cell markers NANOG, CD133 (PROM1), ALDH1A1, and LGR5 are elevated by 1.77-, 2.49-, 2.93-, and 7.32-folds, respectively, after HN-30 cells are treated with the siRNA (Figure 4C). Using gene expression data of HNSCC patient samples downloaded from TCGA, we also found strong negative correlations of the expressions of complex III genes with the expressions of several stem cell markers. In particular, the expressions of CD44 and BMI-1, the aforementioned stem cell markers that are highly elevated by metformin, exhibit the most consistent negative correlation with complex III genes’ expressions (Figure 5A,F). The expressions of stem cell markers or stemness markers c-Met (MET), SLC2A13, PDPN, and ALDH1A3 are also inversely correlated with the expressions of one or more complex III genes (Figure 5B–E) [25]. Collectively, our in vitro and in silico results suggest that lowered complex III activity levels are correlated with higher stemness and CSC presence.

## 3. Discussion

Metformin gained attention as a promising potential anticancer therapy as some studies demonstrated a correlation between metformin use and decreased incidence of cancer, while other studies reported its ability to selectively target CSCs. To date, the CSC-inhibiting ability of metformin has been demonstrated in a variety of tumor types, including breast, pancreatic, lung, skin, and ovarian [3,4,7,26]. However, to the best of our knowledge, this study is the first to test the effects of metformin on HNSCC stem cells. This study is also the first to demonstrate that metformin has negligible effects on the proliferation of a CSC population and even protects against cisplatin. In direct contrast to previous studies, our data suggests that metformin potentiates stem cell genes and self-renewal capabilities in our HNSCC stem cell line, JLO-1. Therefore, the effects of metformin are most likely highly dependent on the tumor cell type, so metformin may not be a viable option for targeting HNSCC stem cells. However, our data do suggest that metformin decreases the proliferation of non-stem HNSCC cells. Several studies have indicated that metformin treatment alone can decrease cancer proliferation using HNSCC cell lines, although each study describes a different mechanism of action, including AMPK-independent downregulation of the mTOR pathway or global inhibition of protein translation [27,28]. These studies are consistent with our data, which indicate that the non-stem cell (ALDH-) fraction of HN-30 decreases in viability after treatment of metformin. Collectively, our results indicate that metformin may be a valuable drug against HNSCC, but only if another drug is used to mitigate its protective effects on HNSCC CSCs. Since metformin is much better tolerated by the body than traditional chemotherapy drugs, it is an attractive therapeutic option that can be used to reduce the amount of chemotherapy drugs needed for the same anti-tumor effects. However, since metformin’s chemoprotection of CSCs will prevent complete elimination of the tumor and render treatment ineffective in the long term, we sought to determine the mechanism with which metformin acts on CSCs to explore the possibility of using a drug to mitigate this effect.

Through computational modelling of metformin’s binding to proteins with the docking software AutoDock Vina, we discovered evidence of a strong binding interaction between metformin and complex III of the mitochondria. Complex III, also known as the cytochrome bc1 complex or coenzyme Q–cytochrome c reductase, is a complex within the electron transport chain of the mitochondria and is known as a major site of ROS production [10,29]. It conducts the Q cycle, in which ubiquinol (QH_2_) is oxidized into ubiquinone (Q, or coenzyme Q). When QH_2_ enters the complex, it binds to the Q_o_ reactive site within the cytochrome b subunit of the complex, where two electrons are extracted from it. One would be transferred to the 2Fe/2S center located within the nearby Rieske protein, while the other would be transferred to the nearby B_L_ heme. The latter electron would flow from the B_L_ heme to the B_H_ heme then to a ubiquinone molecule within the complex, reducing it to the free radical ubisemiquinone, which has been reported to transfer the electron to oxygen, forming ROS [30]. We discovered that metformin binds near the B_L_ heme, suggesting that it is potentially able to block the flow of electrons to ubisemiquinone, thereby preventing the formation of ROS. Indeed, complex III inhibitors that bind near the Q_o_ site, including myxothiazol and stigmatellin, have been demonstrated to reduce the amount of ROS generated by complex III [29,30].

The results of this study could be well-explained under the supposition that metformin inhibits complex III and lowers ROS levels as result. Through qPCR assay of siRNA knocked-down cells and TCGA gene expression correlations, our results suggest that lowered complex III activity correlates with higher stem cell marker expressions and higher histologic grade. Therefore, if metformin inhibits complex III activity, it would be able to induce higher stem cell marker expressions, as was observed in our experiments. Additionally, it is previously known that metformin decreases the amount of ROS in cells, and low ROS levels are essential for the preservation of CSC self-renewal and other pro-survival properties [12,25]. We thus hypothesize that metformin lowers ROS production in complex III to elevate the stemness of HNSCC CSCs and enable their maintenance after application of cisplatin. Moreover, we can also attribute the observed anti-proliferative effect of metformin on non-stem HNSCC cells to metformin’s ability to decrease ROS levels, since elevated ROS levels in cancer cells can drive cancer progression through the activation of HIF-1 [31].

Interestingly, the current literature has not reported that metformin targets complex III. Instead, a well-known potential target of metformin is mitochondrial complex I, a complex upstream of complex III in the electron transport chain that generates the QH_2_ consumed by complex III [6]. However, many outstanding questions and concerns were raised by this hypothesis of complex I inhibition. We thus propose that complex III may be a more direct target of metformin and that this hypothesis could resolve some of the theoretical issues surrounding complex I inhibition by metformin. Many studies have characterized metformin’s inhibition of complex I, but few have provided direct evidence of complex I inhibition due to the difficulties involved in isolating complex I from the mitochondrial membranes [9]. Some labs even claimed failure to directly observe complex I inhibition [9]. Furthermore, no molecular binding site of metformin to complex I has been proposed. The greatest controversy in the theory of complex I inhibition is the fact that extremely high levels of metformin, 1000-fold higher than serum concentration, are needed to induce complex I inhibition [6]. It was proposed that this concentration can be achieved within the mitochondria when driven by the mitochondrial membrane potential, but this hypothesis has not been tested [32]. A recent study that performed whole-body PET scan of radioactive-carbon labeled metformin demonstrated that no such concentration of metformin was reached in head and neck tissues [33]. Additionally, we observed the chemoprotective effects of metformin against CSCs and increased expressions of stem cell genes when only 0.5–0.75 mM of metformin was applied. This is many times lower than the IC50 value (concentration of drug where target activity is inhibited by half) of 19.4 mM reported by one study for metformin inhibition of complex I [32]. According to the dosage-response curve reported, metformin concentration of 0.75 mM would not lead to any inhibition of complex I [32]. Therefore, it is likely that metformin acts on some target other than complex I, such as complex III. One study observed that metformin, unlike the classical complex I inhibitor rotenone, does not lead to an increase in ROS production due to forward electron flux through complex I but only decreases ROS production due to reverse electron flux [11]. The fact that a complex I inhibitor would increase ROS production from forward electron flux and decrease ROS production from reverse electron flux at the same time is more intuitive because when complex I is inhibited, the electron that normally travels through complex I to reduce Q is blocked and would leak out of the complex to react with oxygen, forming ROS. Because the reaction of complex I never occurred, it would be less likely that the reverse reaction would occur where the electron travels backwards to reduce the substrate of complex I. Therefore, the ROS production associated with reverse electron flux would decrease. The fact that metformin only causes a decrease in reverse electron flux may suggest that a downstream target, such as complex III, was inhibited instead of complex I. Finally, it was also reported that biguanide drugs only inhibit complex I when mitochondria are in state 3 (active respiration) but not when they are in state 4 (termination of active respiration), which also hints at the possibility of a target downstream of complex I [34].

Besides exploring the specific molecular mechanism, we have also investigated the effects of metformin on important pathways that are known to significantly influence the CSC phenotype. We demonstrated that metformin induces an increase in the expression of stem cell genes, most notably for CD44 and BMI-1. It is well accepted that BMI-1 is necessary for self-renewal in both cancer and normal stem cells, and CD44 is also one of the most well-known CSC biomarkers [25]. We next explored the effects of metformin on Akt in JLO-1. The PI3K/Akt signaling pathway is frequently dysregulated in cancers, and Akt activation has been shown to contribute to chemotherapeutic resistance [26]. Metformin has been reported to protect glioma cell lines against cisplatin via activation of Akt [5]. However, our results indicate that metformin causes a decrease in Akt levels in CSCs, which implies that the chemo-protective actions of metformin are not conferred through Akt.

Interestingly, we did not observe an increase in tumorsphere formation after application of metformin to JLO-1 (data not shown), most likely because the self-renewal capabilities of the JLO-1 CSCs have already reached the maximum threshold. However, metformin rescues the decrease in cell proliferation caused by cisplatin, suggesting that metformin does not observably affect the activities of normal CSCs and only prevents these cells from being damaged by cisplatin. This observation suggests that cisplatin must act on CSCs in a manner that can be reversed by metformin. One attractive mechanism consistent with our hypotheses would be that cisplatin causes the generation of ROS, which has been reported in some studies, that would cause the CSCs to lose self-renewal capabilities [35,36]. Metformin could then reverse this effect of cisplatin by lowering ROS levels.

In summary, our results suggested that metformin results in the chemoprotection of HNSCC stem cells but decreases the ability of non-stem cancer cells to proliferate. However, we emphasize that our results were purely derived from in vitro and in silico assays and that in vivo experiments are needed to further validate applicability of our results. We propose the novel mechanism that mitochondrial complex III inhibition by metformin causes a reduction in the ROS levels of these cells to yield these observed effects, as low ROS levels are required for stemness, but high ROS levels are drivers of tumor progression. While extensive in vitro binding analyses and functional assays are needed to validate whether complex III is a target of metformin and whether metformin binding to complex III reduces ROS levels, our proposed mechanism raises exciting possibilities for treating HNSCC using metformin in combination with another drug that could mitigates its effects on HNSCC stem cells. One such drug combination can be a molecule that inhibits complex I in CSCs, as it was reported that inhibition of both complex I and complex III of the mitochondria would lead complex II to produce a large amount of ROS [37], which could debilitate HNSCC CSCs.

## 4. Materials and Methods

### 4.1. Cell Lines and Cultures

The JLO-1 cell line was derived from a fresh laryngeal tumor of a patient undergoing tumor resection. A stem cell selective cultivation condition was used to generate JLO-1, as described in our previous study [38]. Briefly, flow cytometry was performed to select for CD44+ cells, which were then grown on laminin-coated plates and cultured in keratinocyte serum-free media (Invitrogen, Carlsbad, CA, USA) containing 2 mM l-glutamine (Invitrogen), 50 μg/mL gentamycin (Invitrogen), and 20 ng/mL EGF and FGF (R&D Systems, Minneapolis, MN, USA), supplemented daily. We also used the HN-30 cell line, a gift from Dr. J.S. Gutkind, University of California San Diego. Cell lines were routinely cultured in DMEM supplemented with 10% fetal bovine serum (FBS), 2% streptomycin sulfate (Invitrogen), and 2% l-glutamine (Invitrogen) and incubated at 37 °C in 5% CO_2_ and 21% O_2_.

### 4.2. FACS Identification of ALDH+ and ALDH- Cell Populations

HN-30 cells were stained with the Aldefluor stem cell detection kit (STEMCELL technologies, Vancouver, BC, Canada), which will lead to fluorescence of cells with high ALDH activity. The ALDH-bright cells were sorted from ALDH-dim cells using a fluorescence-activated flow cytometer.

### 4.3. Cell Proliferation Assay

MTS assays were performed using the CellTiter 96 Aqueous non-radioactive cell proliferation assay (Promega, Madison, WI, USA). Cells were trypsinized, counted, and replated into a 96-well plate at 5000 cells per well. Cells were allowed to adhere overnight. To generate a dose–response curve for cell proliferation vs. metformin concentration, indicated doses of metformin were added to the corresponding wells for an incubation period of 72 h. For synergistic assays involving the combination of cisplatin and metformin, HN-30 cells were treated with 8 or 12 mM metformin for 48 h, followed by co-treatment with cisplatin at a range of doses (1, 2, 5, 10, 20 μM) for an additional 48 h; while JLO-1 cells were treated with 0.5 or 0.7 mM of metformin for 48 h before co-treatment with cisplatin. Each permutation was performed in triplicates. Following the indicated incubation periods for the above assays, 20 μL of the MTS reagent was added into each well followed by a 1–3 h incubation period. The plates were then read at an absorbance of 490 nm.

### 4.4. TUNEL Assay

JLO-1 cells were treated with metformin 4 days prior to fixing in 70% ethanol. Media and growth factors were not replenished throughout the treatment. Using the APO-BRDUTMKit (Phoenix Flow Systems, Inc., San Diego, CA, USA), the cells undergoing apoptosis were labeled with bromolated deoxyuridine triphosphate nucleotides (BrdUTP). These cells were then identified and binded to a fluorescein labeled antiBrdU monoclonal antibody. After the required incubation times, the samples analyzed for the proportion of apoptotic cells by flow cytometry.

### 4.5. Western Blot Analysis

JLO-1 cells were harvested and lysed with lysis buffer containing 20 mM Tris (pH 7.5), 150 mM NaCl, 1 mM ethylenediaminetetraacetic acid (EDTA), 1 mM egtazic acid (EGTA), 1% Triton-x, 2.5 mM sodium pyrophosphate, 1 mM β-glycerophosphate, 1 mM Na_3_VO_4_, and 1 μg/mL leupeptin. Cell lysates were separated on 12% NuPAGE^®^ Novex Bis-Tris Gels (Invitrogen, Carlsbad, CA, USA) and transferred electrophoretically to a PVDF membrane (Immobilon-P membrane, 0.45 μm; Millipore, MA, USA). The membrane was blocked in 5% milk and probed with antibodies for phosphorylated-Akt (p-Akt) (Cell Signaling, Beverly, MA, USA), followed by a secondary antibody. Membranes were visualized with chemiluminescence detection system (Pierce, Rockford, IL, USA). The membranes were probed with antibody against Erk (Abcam, Cambridge, MA, USA) to ensure equal protein loading.

### 4.6. Quantitative Real-Time PCR and siRNA Knockdown

The cultured cells were treated with metformin (0–0.75 mM) for 48 h. Total cell lysate was collected and mRNA was extracted using the RNeasy kit (QIAGEN, Venlo, The Netherlands). cDNA was then synthesized from 1.5 μg of total mRNA using reverse transcriptase (Invitrogen, Carlsbad, CA, USA), as per the manufacturer’s instructions. Real-time quantitative PCR was performed by combining 2.5 μL of the RT with 22.5 μL of SYBR green (Roche, Basel, Switzerland). The reaction was run using System 7300 (Applied Biosystems, Foster City, CA, USA) and results were analyzed by the relative quantity method. Experiments were performed in triplicates with GAPDH expression as the endogenous control. siRNA for the Rieske protein (UQCRFS1) was obtained from Dharmacon, Lafayette, CO, USA. Primers were custom designed by the authors and created by Eurofin Genomics, Louisville, KY, USA. The following sequences were used:

### 4.7. Immunofluorescence

HN-30 cells were cultured on cover slips under 0.75 mM of metformin. The cells were fixed with 4% paraformaldehyde and blocked in goat serum in Dulbecco’s phosphate buffered saline at room temperature prior to incubation with mouse monoclonal to anti-human CD44 (Sigma Aldrich, St. Louis, MO, USA). Cells were then incubated with a goat anti-mouse FITC conjugated secondary antibody (Chemicon, Temecula, CA, USA) and counterstained with DAPI. Finally, SlowFade Gold antifade reagent (Invitrogen, Carlsbad, CA, USA) was used to mount the cover slips onto slides. Fluorescent images were obtained at 40× using the DMIRE2 inverted fluorescence microscope (Leica Microsystems, Buffalo Grove, IL, USA) and computer program Simple PCI (version 6.6, Hamamatsu Photonics, Sewickley, PA, USA) was used for image capture.

### 4.8. Computational Prediction of Metformin Binding Energy

The crystallographic protein structure of mitochondrial complex III was downloaded from the Protein Data Bank (PDB) (www.rcsb.org) under the ID 5OKD, which was contributed by the study of Amporndanai et al. [39]. Metformin molecular structure was downloaded from the ZINC database (http://zinc15.docking.org/). fpocket (version 2.0, University of Paris-Diderot, Paris, France) was used to determine potential binding pockets of complex III, and AutoDock Vina (version 1.1.2, The Scripps Research Institute, San Diego, CA, USA) was used to uncover the position of metformin binding with each pocket that would result in the lowest (most favorable) binding energy [22,40]. The position of binding was then visualized with UCSF chimera (version 1.12, San Francisco, CA, USA) [41]. The favorability of metformin binding was assessed for 250 other proteins most associated with high stemness to determine whether the binding strength of metformin to complex III is relatively large compared to the binding strength of metformin to other binding pockets. To identify the 250 most abundant proteins in tumors with high stemness, we obtained the stemness scores of HNSCC tumors analyzed by TCGA from Malta et. al. [42]. Normalized RNA-seq gene expressions for each HNSCC patient were downloaded from the GDC portal (https://portal.gdc.cancer.gov/) and correlated with these stemness scores using Gene Set Enrichment Analysis (GSEA) (version 3.0, Broad Institute, Inc., Cambridge, MA, USA) [43]. GSEA generated a ranked gene-list that orders genes based on the degree to which expression correlates with stemness scores. The top 250 genes with positive correlation of expression to stemness were chosen, and any available crystallographic structures of proteins and protein complexes associated with these genes were downloaded from PDB. These structures were analyzed with fpocket, which predicted 127,635 binding pockets in total. AutoDock Vina virtual screening was used to determine the lowest possible energy of metformin binding with each pocket. Virtual screening was performed with the Comet supercomputer in the San Diego Supercomputer Center, with access provided through an allocation from the Extreme Science and Engineering Discovery Environment (XSEDE) [44].

### 4.9. Correlation of Complex III Gene Expressions to Survival and Histologic Grade

TCGA mRNA expression read counts downloaded for HNSCC samples were paired with the clinical data of corresponding patients, which include histologic grade and time to death or last follow up. The clinical data were downloaded from the Broad Institute GDAC Firehose (https://gdac.broadinstitute.org/). Survival analyses were performed using the Kaplan-Meier Model, with gene expression designated as a binary variable based on expression above or below the median expression of all samples. Univariate Cox regression analysis was used to identify candidates significantly associated with patient survival (*p* < 0.05). The Kruskal-Wallis test was used to correlate gene expressions to histologic grade (*p* < 0.05). The 11 human genes that encode complex III subunits were included in analysis: MT-CYB, CYC1, UQCRFS1, UQCRC1, UQCRC2, UQCRH, UQCRB, UQCRQ, UQCR9, UQCR10, and UQCR11.

### 4.10. Correlation of Complex III Gene Expressions to Expressions of Stem Cell Markers Using TCGA Data

The expressions of the complex III genes listed above were correlated with the gene expressions of stem cell markers, including CD44, BMI-1, MET, SLC2A13, PDPN, ALDHA3, NANOG, OCT4, etc. The same expression data described in the above section are used. Correlations were assessed using Spearman’s correlation test (*p* < 0.05) and visualized as scatter plots.

## Figures and Tables

**Table 1:** Primer sequences used for quantitative PCR.

CD44	forward:	5′-AGAAGAAAGCCAGTGCGTCT-3′
CD44	reverse:	5′-TGACCTAAGACGGAGGGAGG-3′
GAPDH	forward:	5′-TTCTTTTGCGTCGCCAGCC-3′
GAPDH	reverse:	5′-CGTTCTCAGCCTTGACGGTG-3′
BMI1	forward:	5′-CGAGACAATGGGGATGTGGG-3′
BMI1	reverse:	5′-AAATGAATGCGAGCCAAGCG-3′
ALDH1A1	forward:	5′-CACGCCAGACTTACCTGTCC-3′
ALDH1A1	reverse:	5′-TTGTACGGCCCTGGATCTTG-3′
NANOG	forward:	5′-AATGGTGTGACGCAGGGATG-3′
NANOG	reverse:	5′-ACCTCGCTGATTAGGCTCCA-3′
POU5F1	forward:	5′-TCCCGAATGGAAAGGGGAGA-3′
POU5F1	reverse:	5′-GGCTGAATACCTTCCCAAATAGA-3′
ABCG2	forward:	5′-TTACGCACAGAGCAAAGCCA-3′
ABCG2	reverse:	5′-GCAAGGGGCTAGAAGAAGGG-3′
PROM1	forward:	5′-GAATCCTTTCCATTACGGCGG-3′
PROM1	reverse:	5′-CCTGAAAAGGAGTTCCCGCA-3′
LGR5	forward:	5′-GGAGTTACGTCTTGCGGGAA-3′
LGR5	reverse:	5′-CAGGCCACTGAAACAGCTTG-3′.

## Supplementary Materials

Supplementary materials can be found at http://www.mdpi.com/1422-0067/20/1/193/s1. Figure S1: Western blot assay of phosphorylated-Akt levels after JLO-1 cells were treated with 0–0.75 mM of metformin. Erk level was probed as loading control.

## Abbreviations

CSCs	Cancer stem cells
HNSCC	Head and neck squamous cell carcinoma
ROS	Reactive oxygen species
