# Supplementary figures and images for: Metformin Results in Diametrically Opposed Effects by Targeting Non-Stem Cancer Cells but Protecting Cancer Stem Cells in Head and Neck Squamous Cell Carcinoma
Source: Int J Mol Sci. 2019 Jan 7;20(1):193. doi: 10.3390/ijms20010193 (PMC6337486; doi:10.3390/ijms20010193)

Metformin Concentration (mM)

0

0.25

0.5

0.75

P-Akt

Total Erk

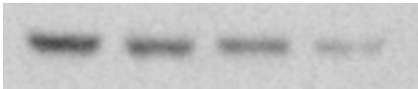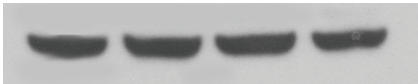

Supplement: Supplementary file 1 [file ijms-20-00193-s001.pdf]
